# Supplementary material for: Mouse enteric neurons control intestinal plasmacytoid dendritic cell function via serotonin-HTR7 signaling
Source: Nat Commun. 2024 Oct 25;15:9237. doi: 10.1038/s41467-024-53545-2 (PMC11511829; doi:10.1038/s41467-024-53545-2)
Supplement: Supplementary file 1 — Supplementary info [file 41467_2024_53545_MOESM1_ESM.pdf]

Mouse enteric neurons control intestinal plasmacytoid dendritic cell function via serotonin-HTR7 signaling

Supplementary information

Hailong Zhang[1,2,3], Yuko Hasegawa[1,2,3], Masataka Suzuki[1,2,3], Ting Zhang[1,2,3], Deborah R. Leitner[1,2,3], Ruaidhrí P. Jackson[4], Matthew K. Waldor[1,2,3]*

Contents

Supplementary figures

Supplementary tables

Reference

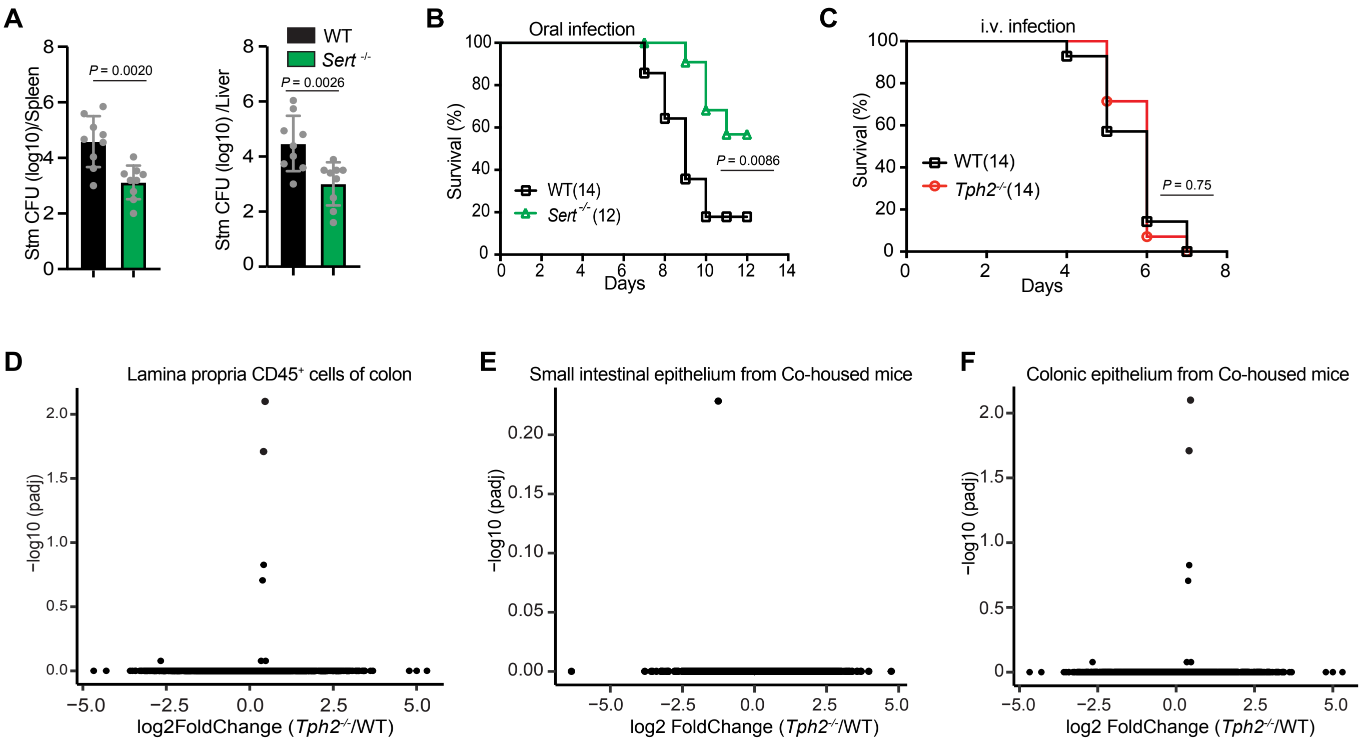

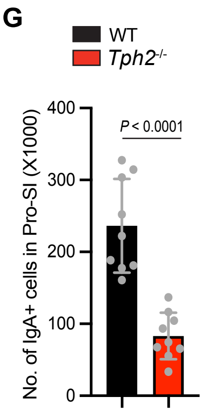

Supplementary Figure 1. Neuronal serotonin does not have a major impact on gene expression in the intestinal epithelium.

(A) *Salmonella* Typhimurium (STm) CFU burdens in spleens and livers of WT and *Sert*$^{-/-}$ mice 5 days post oral inoculation (WT, n = 9; *Sert*$^{-/-}$, n = 9).

(B) Kaplan Meier plot of survival of WT and *Sert*$^{-/-}$ mice following oral STm inoculation (WT, n = 14; *Sert*$^{-/-}$, n = 12).

(C) Kaplan Meier plot of survival of WT and *Tph2*$^{-/-}$ mice after intravenous STm infection (n = 14).

(D-F) Comparison of gene expression in LP CD45+ cells from the colon (D), and epithelium from the small intestine (E) and colon (F) of co-housed WT and *Tph2*$^{-/-}$ mice.

(G) Total number of IgA positive B cells isolated from proximal small intestine in mice of the indicated genotype (n = 9).

Data shown are means ± SD. Statistical analysis was performed by a two-tailed Mann-Whitney test in A and G and by a Log-rank test in B and C.

Figure S1

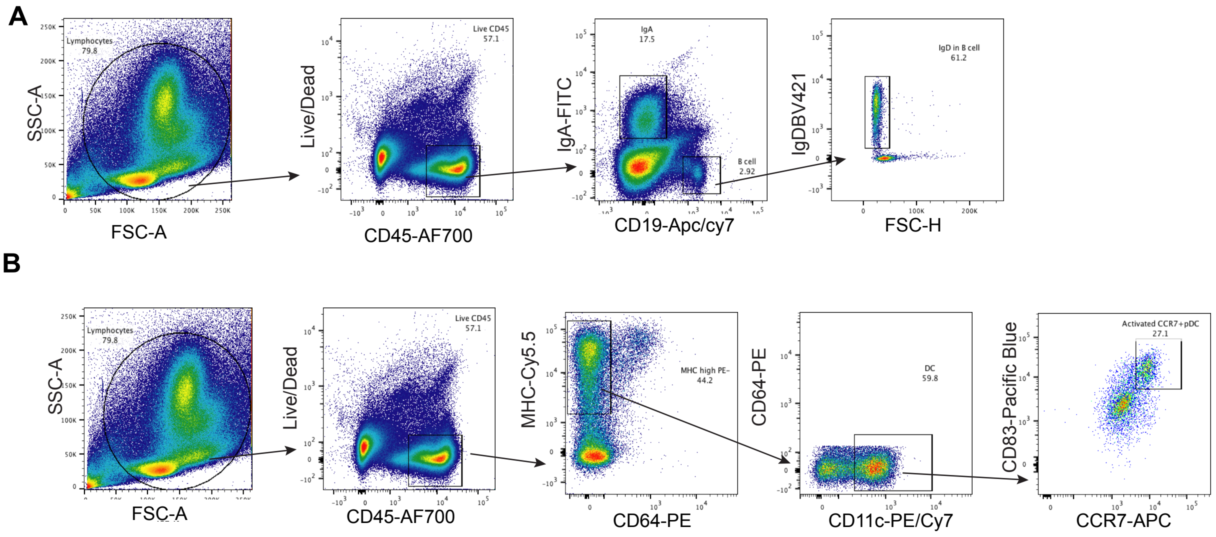

Supplementary Figure 2. Gating strategy for detection and sorting of different immune cell types.
(A) B cells were gated as live CD45+ CD19+; IgD cells were gated as live CD45+ CD19+ IgD+; IgA cells were gated as live CD45+ IgA+ related to figure 1E, 2F.
(B) DCs were gated as live CD45+ MHCII+ CD64- CD11c+ related to figure 3C, D and 4H-J.

Figure S2

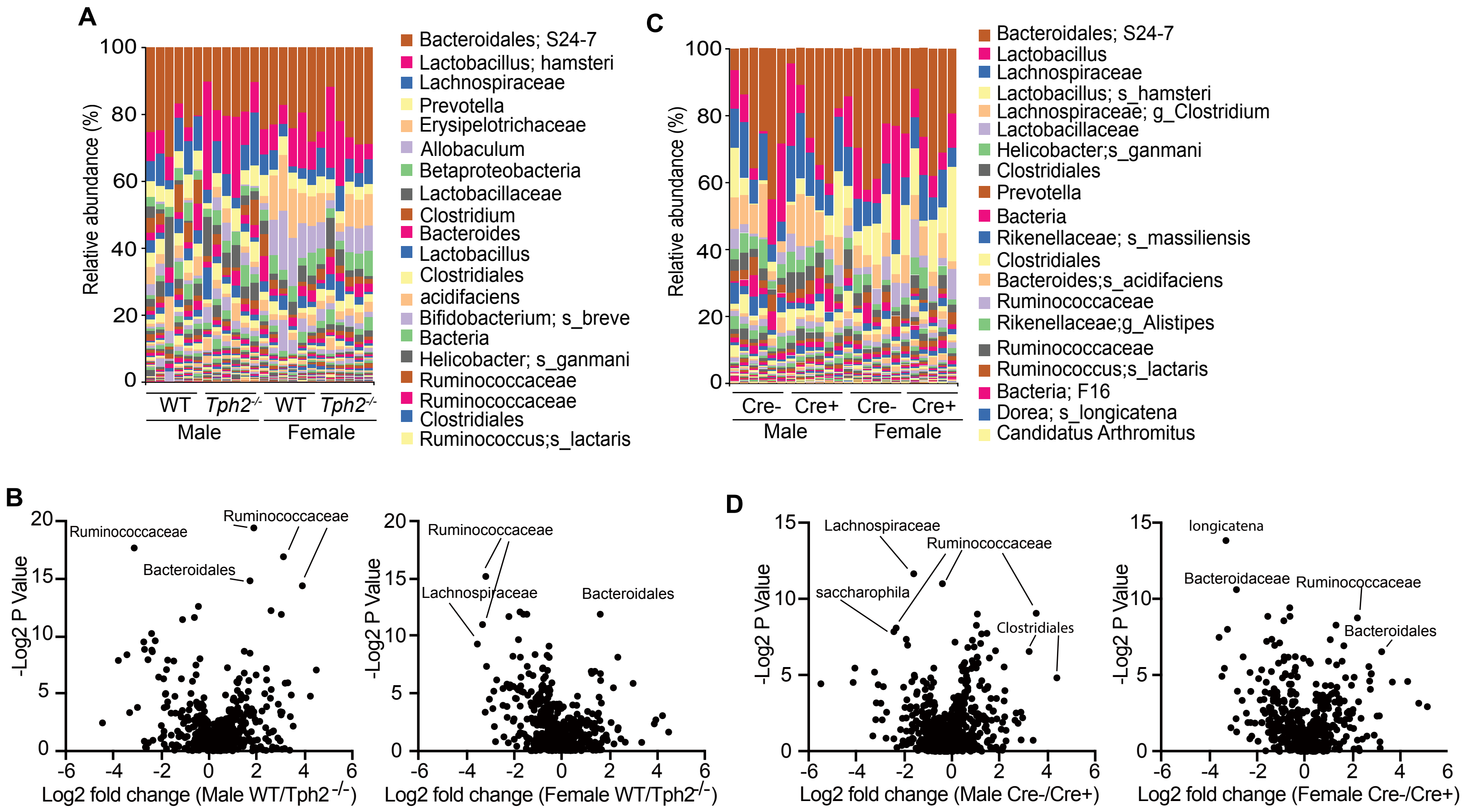

Supplementary Figure 3. Neuronal serotonin does not have a major impact on the composition of the microbiota.

(A, C) Bar plot of microbiota taxa at the genus and species level in mice of the indicated genotype (n = 6). (B, D) Differential abundance of fecal microbiota at the OTU level between WT and *Tph2* KO animals (B), as well as between Hand2 Cre-positive and negative animals (D).

Figure S3

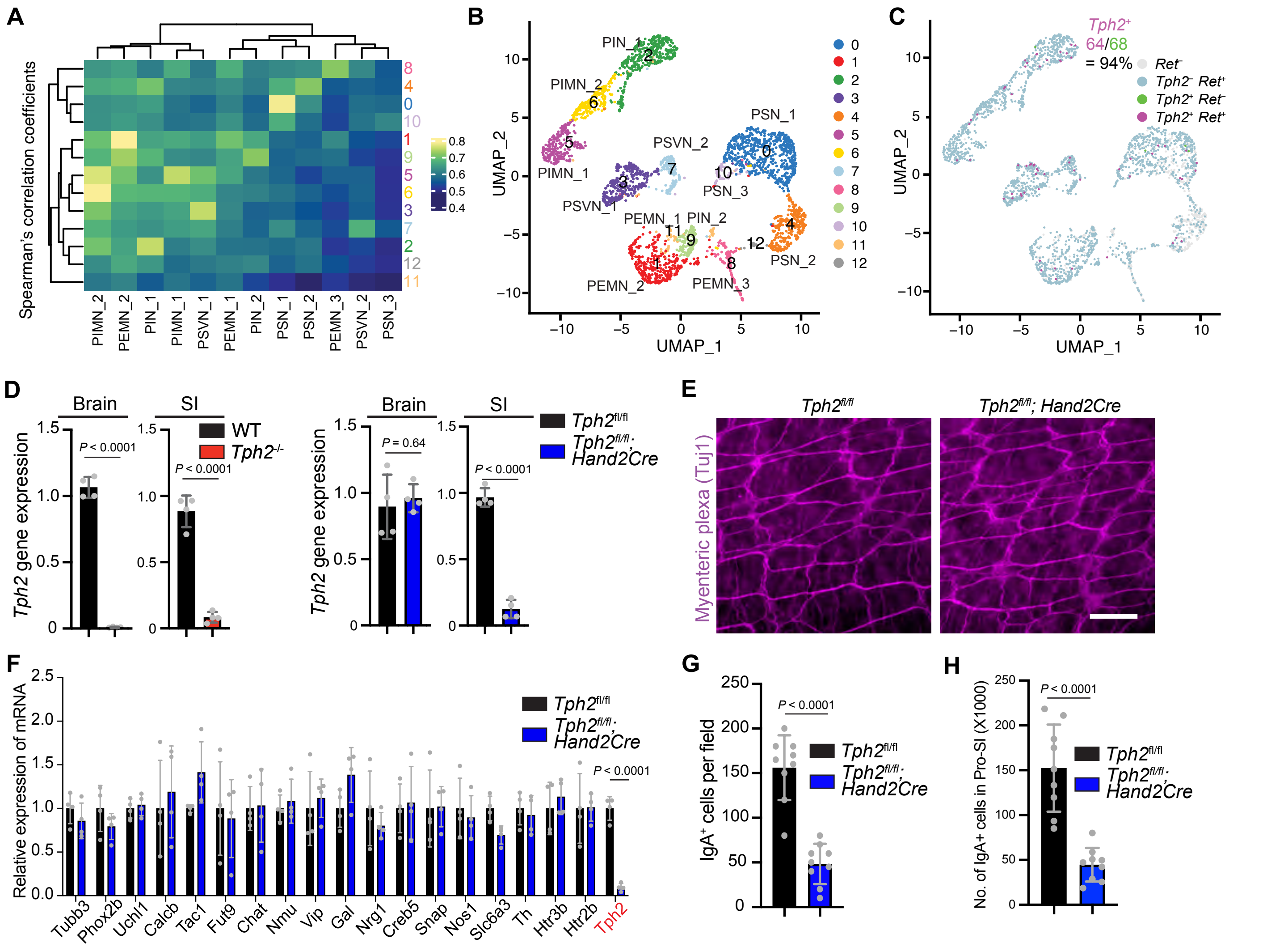

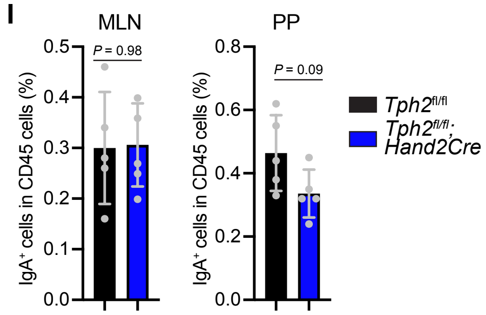

Supplementary Figure 4. The structure and gene expression in the small intestine myenteric plex are similar in *Tph2*$^{fl/fl}$ and *Tph2*$^{fl/fl}$; Hand2-Cre mice.

(A-B) Comparison of enteric neuron subtypes detected in this study with those in[1]. The color scale in the heatmap in (A) represents Spearman's rank correlation coefficients between the cell clusters found in this study and the reference cell types in Drokhlyansky et[1]. The UMAP in (B) shows the correspondence of the clusters found in this study with those in Drokhlyansky et[1].

(C) UMAP shows the majority of *Tph2*+ cells are *Ret*+ enteric neurons.

(D) RT-qPCR analysis of *Tph2* gene expression in the brainstem and the muscularis propria of the small intestine from mice of the indicated genotype (n = 4 mice/group).

(E) Immunofluorescent staining of the small intestine myenteric plexus with AF647 conjugated anti-Tuj1 antibody; scale bar, 100 µm.

(F) RT-qPCR analyses of the expression of neuronal marker genes in the small intestine muscularis propria from *Tph2*$^{fl/fl}$ and *Tph2*$^{fl/fl}$; Hand2-Cre mice (n = 4 mice/group).

(G) Quantification of IgA+ cells per field from nine fields per group (n = 3 mice/group).

(H) Total number of IgA+ B cells isolated from proximal small intestine in mice of the indicated genotype (n = 9).

(I) Flow cytometry analysis of the frequency of IgA+ cells in CD45+ cells from the MLN and PP from co-housed *Tph2*$^{fl/fl}$ and *Tph2*$^{fl/fl}$; Hand2-Cre mice (n = 5).

Data shown are means ± SD. Statistical analysis was performed by two-tailed unpaired Student's *t*-test in D, F and by two-tailed Mann-Whitney test in G, H, I.

Figure S4

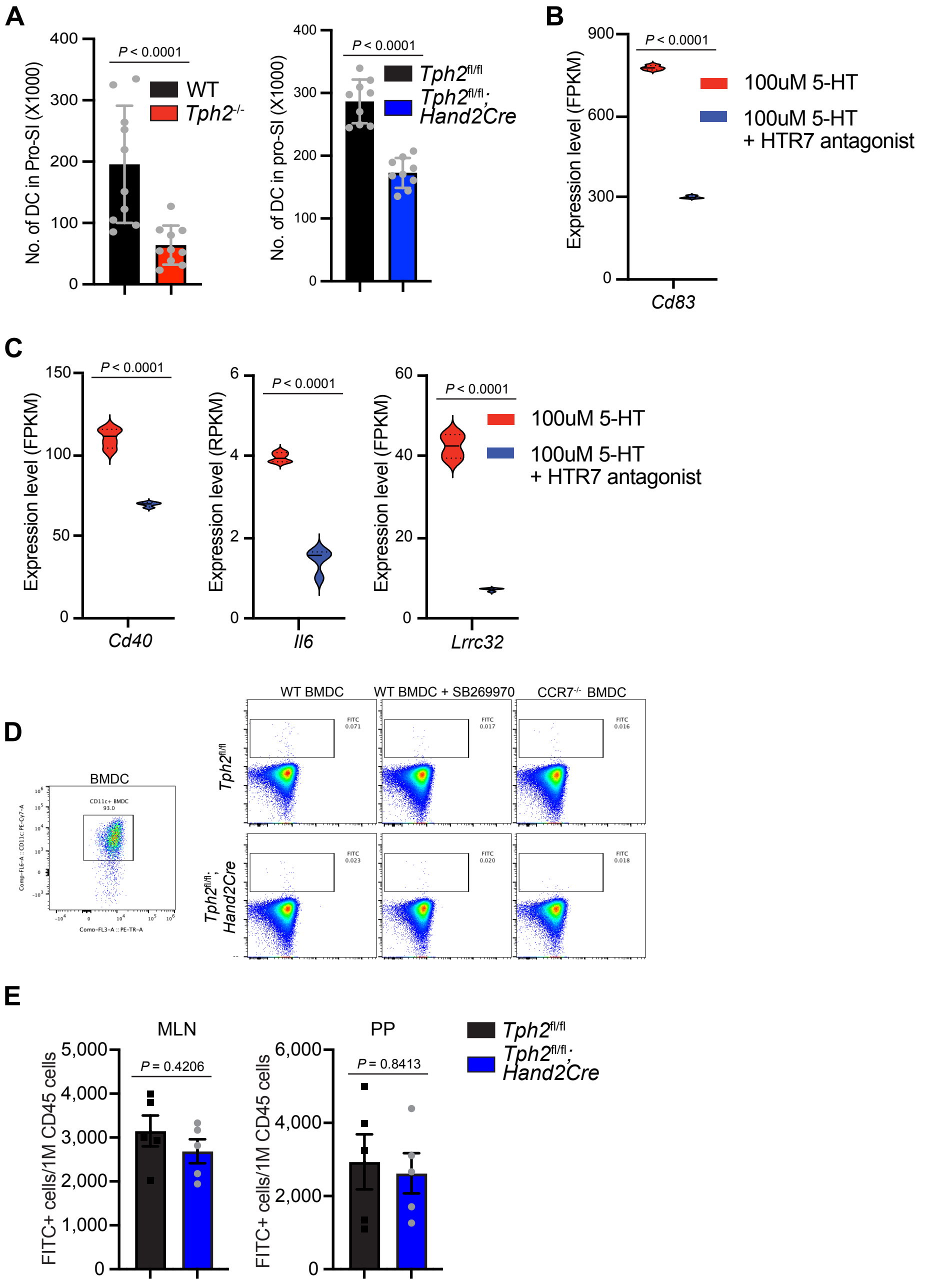

Supplementary Figure 5. Serotonin regulates LPDC gene expression through the 5-HT receptor 7.
(A) Total number of DC isolated from proximal small intestine in mice of the indicated genotype (n = 9).
(B-C) Expression of select genes in LPDCs after treatment with serotonin ± HTR7 antagonist (SB269970) (n = 4).
(D) Representative flow cytometry plots showing the proportion of CFSE+ BMDC homing to the lamina propria of the small intestine in *Tph2*$^{fl/fl}$ and *Tph2*$^{fl/fl}$; Hand2-Cre mice.
(E) Frequency of CFSE+ BMDC among CD45+ cells in the MLN and PP of *Tph2*$^{fl/fl}$ and *Tph2*$^{fl/fl}$; Hand2-Cre mice (n = 5).
Data shown are means ± SD. Statistical analysis was performed by two-tailed Mann-Whitney test in A and E, by two-tailed unpaired Student's *t*-test in B and C.

Figure S5

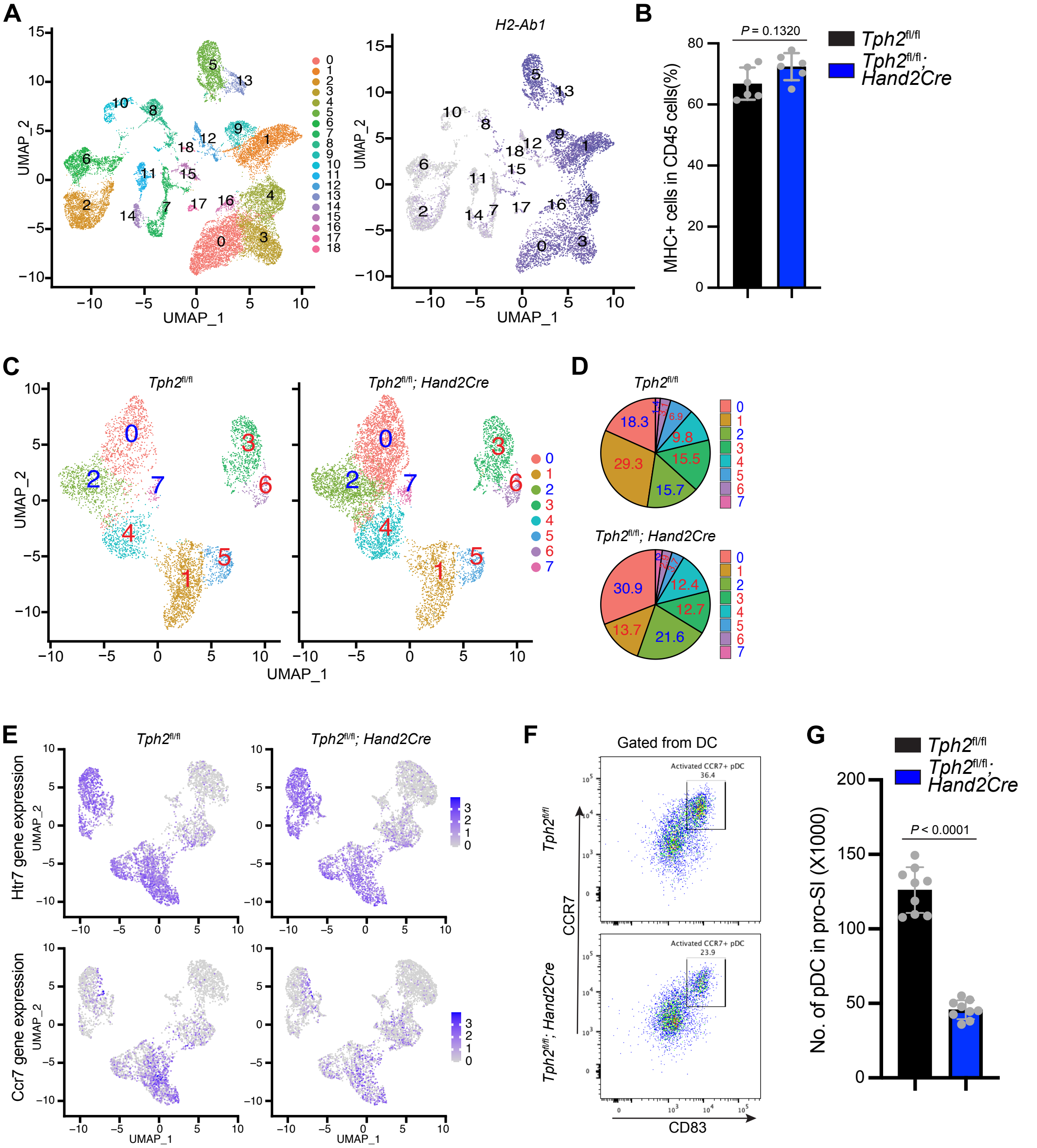

Supplementary Figure 6. ESN remodel LPDC function *in vivo*.

(A) UMAP plot of single-cell transcriptome data of cells by DC selected from the small intestine lamina propria from *Tph2*$^{fl/fl}$ mice. Clusters 2, 6-8, 10-12, 14, 15, 17, and 18, which did not express *H2-Ab1* (MHC-II) were computationally removed to create the UMAP in C.

(B) Flow cytometry analysis of the frequency of MHCII+ cells in CD45+ cells from the small intestine lamina propria of co-housed *Tph2*$^{fl/fl}$ and *Tph2*$^{fl/fl}$; Hand2-Cre mice (n = 6).

(C) UMAP plots comparing the abundance of MHC-II positive clusters of DC in *Tph2*$^{fl/fl}$; Hand2- Cre compared with *Tph2*$^{fl/fl}$ mice. The conditional KO animals had decreased proportions of DC (clusters 1, 3, 4, 5, and 6 defined by CD11c+).

(D) Pie charts show decreased proportions of LPDC (Cluster 1, 3, 4, 5, and 6) in *Tph2*$^{fl/fl}$; Hand2- Cre compared with *Tph2*$^{fl/fl}$ mice (64.6% vs 45.5%).

(E) UMAP plot derived from single-cell transcriptome data showing the expression of *Htr7* and *Ccr7* in LPDCs from *Tph2*$^{fl/fl}$ and *Tph2*$^{fl/fl}$; Hand2-Cre mice. The color scale represents relative expression level.

(F) Representative flow cytometry plots showing the proportion of CCR7+ CD83+ DC in the lamina propria of the small intestine in *Tph2*$^{fl/fl}$ and *Tph2*$^{fl/fl}$; Hand2-Cre mice.

(G) Total number of pDC isolated from proximal small intestine in *Tph2*$^{fl/fl}$ and *Tph2*$^{fl/fl}$; Hand2- Cre mice (n = 8). Data shown are means ± SD. Statistical analysis was performed by two-tailed Mann-Whitney test in B and G.

Figure S6

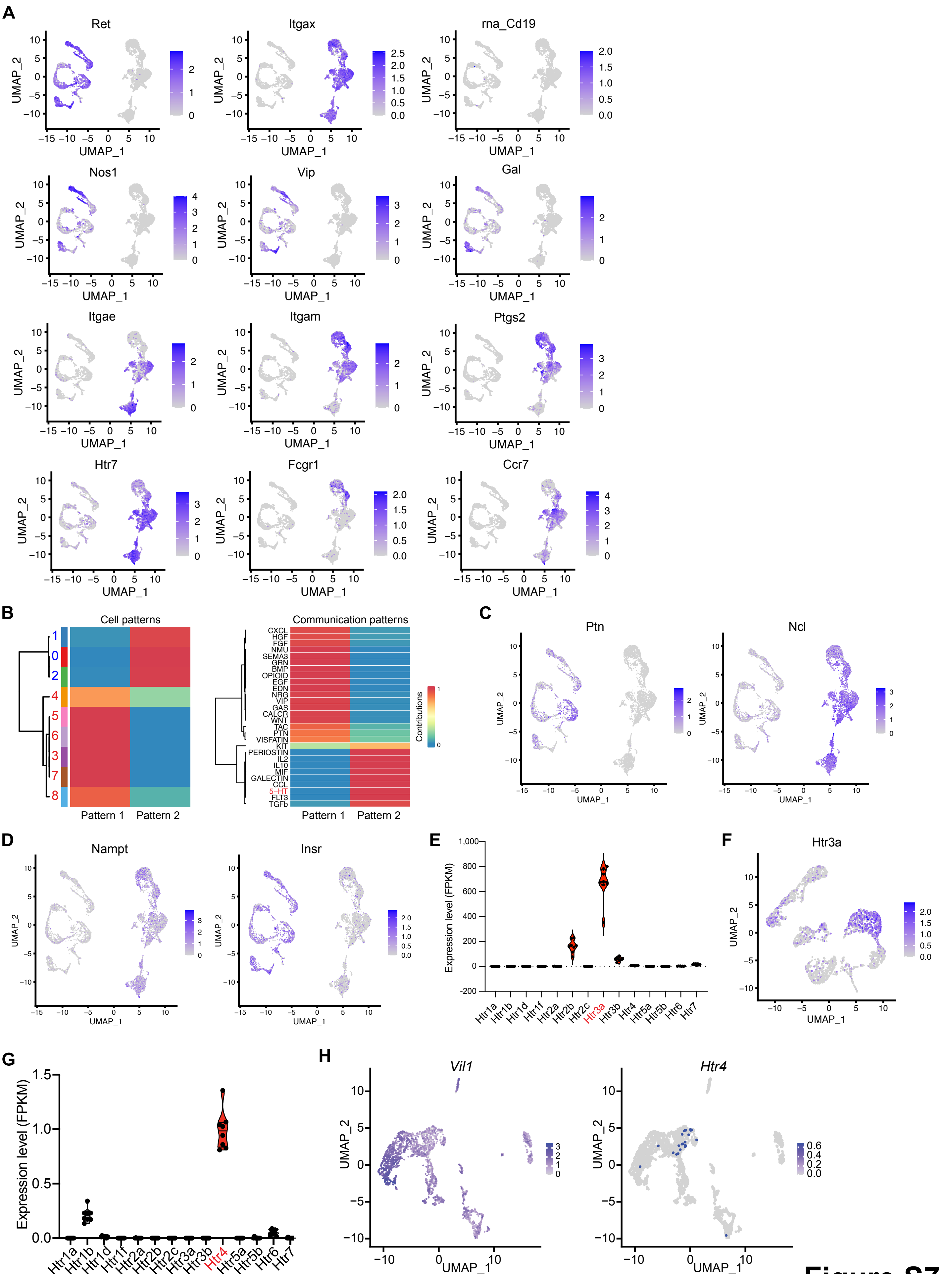
A
Ret
Itgax
rna_Cd19
Nos1
Vip
Gal
Itgae
Itgam
Ptgs2
Htr7
Fcgr1
Ccr7
UMAP_1
UMAP_2
B
Cell patterns
Communication patterns
CXCL
HGF
FGF
NMU
SEMA3
GRN
BMP
OPIOID
EGF
EDN
NRG
VIP
GAS
CALCR
WNT
TAC
PTN
VISFATIN
KIT
PERIOSTIN
IL2
IL10
MIF
GALECTIN
CCL
5-HT
FLT3
TGFb
Contributions
Pattern 1
Pattern 2
C
Ptn
Ncl
D
Nampt
Insr
E
Expression level (FPKM)
Htr1a
Htr1b
Htr1d
Htr1f
Htr2a
Htr2b
Htr2c
Htr3a
Htr3b
Htr4
Htr5a
Htr5b
Htr6
Htr7
F
Htr3a
G
Expression level (FPKM)
H
Vil1
Htr4

Figure S7

Supplementary Figure 7. Bidirectional communication between enteric neurons and LPDC.
(A) UMAP plots derived from single-cell transcriptome data showing expression of indicated genes in enteric neurons (left population) and LPDC (right population).
(B) Heatmap showing the two global communication patterns calculated by the key signals [2] for subpopulations between enteric neurons and LPDC.
(C) UMAP plot derived from single-cell transcriptome data showing expression of *Ptn* in enteric neurons and its receptor *Ncl* in LPDC.
(D) UMAP plot derived from single-cell transcriptome data showing expression of *Nampt* in LPDC and its receptor *Insr* in enteric neurons.
(E) *Htr3a* is the dominant serotonin receptor in enteric neurons. FPKM (Fragments Per Kilobase of transcript per Million fragments mapped) values of all serotonin receptors in enteric neurons.
(F) UMAP plot derived from single-cell transcriptome data showing expression of *Htr3a* in enteric putative sensory neurons.
(G) *Htr4* is the dominant serotonin receptor in small intestinal epithelial cells. FPKM (Fragments Per Kilobase of transcript per Million fragments mapped) values of all serotonin receptors in small intestinal epithelial cells.
(H) UMAP plot derived from single-cell transcriptome data in Tsang et al [3] showing expression of *Htr4* in epithelial cells (Vil1+) in the small intestine.

Figure S7

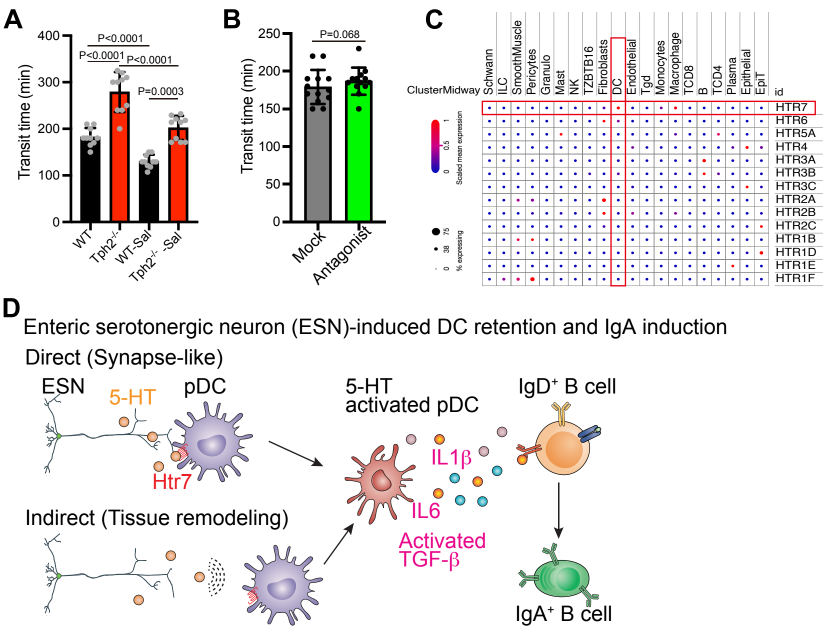

Supplementary Figure 8. Schematic of ESN-DC interaction.

(A) Total GI transit time was measured using a carmine red assay in WT and *Tph2*$^{-/-}$ before and after STm infection (n = 10 mice per group).

(B) Gut transit time in mock (PBS) and HTR7 antagonist-treated mice. The mice were treated with ip PBS or an HTR7 antagonist (SB269970, 10 mg/kg) 15 minutes prior to the measurement of gut transit time with carmine red assay.

(C) Expression of differential serotonin receptors in the human colon cancer atlas. The data was visualized using Single Cell Portal with the parameters: clustering (all cells), annotation (Cluster-MidWay), subsampling (all cells), and gene collapse (none). This analysis reveals a proportion of human dendritic cells express *Htr7* mRNA.

(D) Serotonin secreted by enteric serotonergic neurons activates LPDC through the 5-HT receptor 7 via a synapse-like mechanism or an indirect pathway that leads to increased expression of genes crucial for DC-mediated differentiation of IgA+ B cells from precursor IgD+ cells.

Figure S8

Supplementary Table 1

This table contains detailed information about all the antibodies (1:200 dilution) used in this study.

ANTIBODIES	SOURCE	IDENTIFIER
Purified anti-mouse CD16/32 Antibody	BioLegend	Cat# 101302, RRID:AB_312801
Alexa Fluor® 700 Rat Anti-Mouse CD45	BioLegend	Cat# 103128 RRID:AB_493715
Alexa Fluor® 488 Rat Anti-Mouse I-A/I-E	BD Biosciences	Cat# 562352, RRID:AB_11151902
PE Mouse anti-Mouse CD64 a and b Alloantigens	BD Biosciences	Cat# 558455, RRID:AB_647241
PE-Cy™7 Hamster Anti-Mouse CD11c	BD Biosciences	Cat# 558079 RRID:AB_647251
CD8 alpha Monoclonal Antibody (5H10), PE-Texas Red	Thermo Fisher Scientific	Cat# MCD0817 RRID:AB_10374589
APC-Cy™7 Hamster Anti-Mouse CD3e	BD Biosciences	Cat# 557596 RRID:AB_396759
FITC Rat Anti-Mouse IgA	BD Biosciences	Cat# 559354, RRID:AB_397235
PerCP-Cy™5.5 Rat Anti-Mouse CD45R/B220	BD Biosciences	Cat# 552771, RRID:AB_394457
BV421 Rat Anti-Mouse IgD	BD Biosciences	Cat# 744291, RRID:AB_2742121
PerCP/Cyanine5.5 anti-mouse CD326 (Ep-CAM)	BioLegend	Cat# 118219, RRID:AB_2098647
Alexa Fluor(R) 647 anti-Tubulin beta 3	BioLegend	Cat# 801210, RRID:AB_2686931
Purified (azide-free) F(ab')2 Goat anti-mouse IgM (u chain)	BioLegend	Cat# 157102, RRID:AB_2814087
APC anti-mouse CD197 (CCR7) Antibody	BioLegend	Cat# 120108, RRID: AB_389234
Alexa Fluor 488 Rat Anti-Mouse CD83	BD Biosciences	Cat# 563539, RRID: AB_2738268
AF488 Rabbit 5-HT7 Polyclonal Antibody	My BioSource	Cat# MBS9455759

Supplementary Table 2

The list of primers used for real-time quantitative PCR analyses in this study.

Gene	Forward primer	Reverse Primer	Species
Gapdh	AGG TCG GTG TGA ACG GAT TTG	TGT AGA CCA TGT AGT TGA GGT CA	Mouse
Tubb3	TAG ACC CCA GCG GCA ACT AT	GTT CCA GGT TCC AAG TCC ACC	Mouse
Phox2b	GGG CTA AGT TTC GCA AGC AG	CAG TGC TGT CGG GAT CAG TG	Mouse
Uchl1	GAT GCT GAA CAA AGT GTT GGC	GGA GTT TCC GAT GGT CTG CTT	Mouse
Calcb	CTC TCA GCA CGA TAT GGG TCC	GCA AGA GAT GTT TTT CCT GGT CG	Mouse
Tac1	AAG CGG GAT GCT GAT TCC TC	TCT TTC GTA GTT CTG CAT TGC G	Mouse
Fut9	TCG CCC ATT TCT AAT CGT CTG C	AGA CTC CAT TGG ACT GAA GAC C	Mouse
Chat	CCA TTG TGA AGC GGT TTG GG	GCC AGG CGG TTG TTT AGA TAC A	Mouse
Nmu	GAG GGA GCT TTG CCG TAT AGT	GAT GCA CAA CAG AGG ACA CAA	Mouse
Vip	AGT GTG CTG TTC TCT CAG TCG	GCC ATT TTC TGC TAA GGG ATT CT	Mouse
Gal	GGC AGC GTT ATC CTG CTA GG	CTG TTC AGG GTC CAA CCT CT	Mouse
Nrg1	ATG GAG ATT TAT CCC CCA GAC A	GTT GAG GCA CCC TCT GAG AC	Mouse
Creb5	AGG ATC TTC TGC CGT CTT GAT	GCG CAG CCT TCA GTC TCA T	Mouse
Snap25	CAA CTG GAA CGC ATT GAG GAA	GGC CAC TAC TCC ATC CTG ATT AT	Mouse
Nos1	CTG GTG AAG GAA CGG GTC AG	CCG ATC ATT GAC GGC GAG AAT	Mouse
Slc6a3	Mm00438388_m1		Mouse
Th	Mm00447557_m1		Mouse
Htr3b	CTG TCT ACC TGG ACC TTT GCG	AAC TCA TCG TTC CAA ACC TCT C	Mouse
Htr4	AGT TCC AAC GAG GGT TTC AGG	CAG CAG GTT GCC CAA GAT G	Mouse
Htr2b	GAA CAA AGC ACA ACT TCT GAG C	CCG CGA GTA TCA GGA GAG C	Mouse
Tph2	GGT TGT CCT TGG ATT CTG CTG	GCC TGG ATT CGA TAT GAA GCA T	Mouse

Reference

1 Drokhlyansky, E. *et al.* The Human and Mouse Enteric Nervous System at Single-Cell Resolution. *Cell* **182**, 1606-1622 e1623, doi:10.1016/j.cell.2020.08.003 (2020).

2 Jin, S. *et al.* Inference and analysis of cell-cell communication using CellChat. *Nat Commun* **12**, 1088, doi:10.1038/s41467-021-21246-9 (2021).

3 Tsang, D. K. L. *et al.* A single cell survey of the microbial impacts on the mouse small intestinal epithelium. *Gut Microbes* **14**, 2108281, doi:10.1080/19490976.2022.2108281 (2022).
