# Supplementary material for: Mouse enteric neurons control intestinal plasmacytoid dendritic cell function via serotonin-HTR7 signaling
Source: Nat Commun. 2024 Oct 25;15:9237. doi: 10.1038/s41467-024-53545-2 (PMC11511829; doi:10.1038/s41467-024-53545-2)
Supplement: Supplementary file 3 — Description of Additional Supplementary Files [file 41467_2024_53545_MOESM3_ESM.pdf]

Description of Additional Supplementary Files

File Name: Supplementary Movie 1.

Description: This video shows enteric serotonergic neurons in the villus.
