# Supplementary material for: Mouse enteric neurons control intestinal plasmacytoid dendritic cell function via serotonin-HTR7 signaling
Source: Nat Commun. 2024 Oct 25;15:9237. doi: 10.1038/s41467-024-53545-2 (PMC11511829; doi:10.1038/s41467-024-53545-2)
Supplement: Supplementary file 5 — Reporting Summary [file 41467_2024_53545_MOESM5_ESM.pdf]

nature portfolio

Corresponding author(s): Matthew K. Waldor

Last updated by author(s): Mar 21, 2024

Reporting Summary

Nature Portfolio wishes to improve the reproducibility of the work that we publish. This form provides structure for consistency and transparency in reporting. For further information on Nature Portfolio policies, see our Editorial Policies and the Editorial Policy Checklist.

Statistics

For all statistical analyses, confirm that the following items are present in the figure legend, table legend, main text, or Methods section.

n/a	Confirmed	
☐	☒	The exact sample size (n) for each experimental group/condition, given as a discrete number and unit of measurement
☐	☒	A statement on whether measurements were taken from distinct samples or whether the same sample was measured repeatedly
☐	☒	The statistical test(s) used AND whether they are one- or two-sided<br>*Only common tests should be described solely by name; describe more complex techniques in the Methods section.*
☐	☒	A description of all covariates tested
☐	☒	A description of any assumptions or corrections, such as tests of normality and adjustment for multiple comparisons
☐	☒	A full description of the statistical parameters including central tendency (e.g. means) or other basic estimates (e.g. regression coefficient) AND variation (e.g. standard deviation) or associated estimates of uncertainty (e.g. confidence intervals)
☐	☒	For null hypothesis testing, the test statistic (e.g. F, t, r) with confidence intervals, effect sizes, degrees of freedom and P value noted<br>*Give P values as exact values whenever suitable.*
☒	☐	For Bayesian analysis, information on the choice of priors and Markov chain Monte Carlo settings
☒	☐	For hierarchical and complex designs, identification of the appropriate level for tests and full reporting of outcomes
☐	☒	Estimates of effect sizes (e.g. Cohen's d, Pearson's r), indicating how they were calculated

Our web collection on statistics for biologists contains articles on many of the points above.

Software and code

Policy information about availability of computer code

Data collection	FAC Symphony analyzer, Eclipse Ti confocal microscope, Miseq, Nextseq 500
Data analysis	FlowJo version 10, FIJI Is Just Image J version 2, QIIME version 1.9.1, GraphPad Prism5

For manuscripts utilizing custom algorithms or software that are central to the research but not yet described in published literature, software must be made available to editors and reviewers. We strongly encourage code deposition in a community repository (e.g. GitHub). See the Nature Portfolio guidelines for submitting code & software for further information.

Data

Policy information about availability of data

All manuscripts must include a data availability statement. This statement should provide the following information, where applicable:

- Accession codes, unique identifiers, or web links for publicly available datasets
- A description of any restrictions on data availability
- For clinical datasets or third party data, please ensure that the statement adheres to our policy

All scRNA-seq, bulk RNA-seq datasets and 16S sequencing data generated here have been deposited into the NCBI Gene Expression Omnibus database under accession number GSE227340.

Research involving human participants, their data, or biological material

Policy information about studies with human participants or human data. See also policy information about sex, gender (identity/presentation), and sexual orientation and race, ethnicity and racism.

Reporting on sex and gender	NA
Reporting on race, ethnicity, or other socially relevant groupings	NA
Population characteristics	NA
Recruitment	NA
Ethics oversight	NA

Note that full information on the approval of the study protocol must also be provided in the manuscript.

Field-specific reporting

Please select the one below that is the best fit for your research. If you are not sure, read the appropriate sections before making your selection.

[x] Life sciences [ ] Behavioural & social sciences [ ] Ecological, evolutionary & environmental sciences

For a reference copy of the document with all sections, see nature.com/documents/nr-reporting-summary-flat.pdf

Life sciences study design

All studies must disclose on these points even when the disclosure is negative.

Sample size	The sample size was indicated in the figure legends.
Data exclusions	NA
Replication	At least three biological replicates were preformed as indicated.
Randomization	All animals were randomly distributed into different cages, with both WT and KO specimens, to minimize microbiota differences.
Blinding	The results were blinded to the investigator until the final analysis.

Reporting for specific materials, systems and methods

We require information from authors about some types of materials, experimental systems and methods used in many studies. Here, indicate whether each material, system or method listed is relevant to your study. If you are not sure if a list item applies to your research, read the appropriate section before selecting a response.

Materials & experimental systems

n/a	Involved in the study	
[ ]	[x]	Antibodies
[ ]	[x]	Eukaryotic cell lines
[x]	[ ]	Palaeontology and archaeology
[ ]	[x]	Animals and other organisms
[x]	[ ]	Clinical data
[x]	[ ]	Dual use research of concern
[x]	[ ]	Plants

Methods

n/a	Involved in the study	
[x]	[ ]	ChIP-seq
[ ]	[x]	Flow cytometry
[x]	[ ]	MRI-based neuroimaging

Antibodies

Antibodies used	CD45 (30-F11), MHC-II (M5/114.15.2), CD64 (X54-5/7.1), CD11c (HL3), CCR7 (4B12), CD83 (Michel-19), B220 (RA3-6B2), IgD (11-26c), and IgA (C10-3) all from Becton Dickson. Anti-HTR7 polyclonal antibody is from MyBiosource.
Validation	All antibodies used in this study are commercially available and have been validated by the vendor.

Eukaryotic cell lines

Policy information about cell lines and Sex and Gender in Research

Cell line source(s)	The B16-FLT3L cell line was obtained from Dr. Ulrich H. Von Andrian at Harvard Medical School. All other cells used were primary cells isolated from mice.
Authentication	NA
Mycoplasma contamination	NO
Commonly misidentified lines (See ICLAC register)	NO

Animals and other research organisms

Policy information about studies involving animals; ARRIVE guidelines recommended for reporting animal research, and Sex and Gender in Research

Laboratory animals	C57BL/6, Tg(Uchl1-HISTH2BE/mCherry/EGFP*)Fsout/J, CD11c-DTR/GFP, Sert-/-, Ccr7-/- and Tph2flox/flox mice were purchased from the Jackson Laboratory (Bar Harbor, ME, USA); Tph2-/- mice were a generous gift from Dr. Gerard Karsenty (Columbia University, NY, USA); Hand2-Cre transgenic mice were a generous gift from Ruaidhrí Jackson (Harvard Medical School, MA, USA). Tph2flox/flox mice were backcrossed to C57BL/6J background for at least six generations. All mice were maintained on a 12-hour light/dark cycle and a standard chow diet at the Harvard Institute of Medicine specific pathogen-free (SPF) animal facility.
Wild animals	NO
Reporting on sex	Sex-matched animals were used in this project.
Field-collected samples	NO
Ethics oversight	Animal experiments were performed according to guidelines from the Center for Animal Resources and Comparative Medicine at Harvard Medical School. All protocols and experimental plans were approved by the Brigham and Women's Hospital Institutional Animal Care and Use Committee (Protocol #2016N000416).

Note that full information on the approval of the study protocol must also be provided in the manuscript.

Flow Cytometry

Plots

Confirm that:

☒ The axis labels state the marker and fluorochrome used (e.g. CD4-FITC).

☒ The axis scales are clearly visible. Include numbers along axes only for bottom left plot of group (a 'group' is an analysis of identical markers).

☒ All plots are contour plots with outliers or pseudocolor plots.

☒ A numerical value for number of cells or percentage (with statistics) is provided.

Methodology

Sample preparation	To isolate lamina propria lymphocytes, the epithelium-depleted intestinal tissues were washed in RPMI medium with 5% (vol/vol) FBS, further minced into small pieces, and then digested by 250 rpm stirring at 37°C in RPMI medium containing collagenase D (0.5 mg/ml, 11088866001, Sigma), Dispase II (0.5 mg/ml, 17105041, Gibco), DNase I (50 ug/ml, 10104159001, Roche) and 5% (vol/vol) FBS for 40 min. The digested tissues were filtered, and lamina propria cells were collected by centrifugation. The pellets were resuspended, and the lamina propria lymphocytes were isolated by Percoll (40%/80%, GE17-0891-01, GE Healthcare) gradient centrifugation. Mesenteric lymph nodes and Peyer's patches were mechanically disrupted. The purified immune cells were used for FACS analysis and sorting. The sorted CD45+ cells were used for bulk RNA-seq analysis.
Instrument	Stained cells were analyzed with a FACSymphony analyzer (Becton Dickson) or sorted on an SH800 Cell Sorter.
Software	Analyses were performed with FlowJo software (v10.8).
Cell population abundance	The sorted cells were analyzed using a FACS analyzer, with a purity exceeding 95%.
Gating strategy	The gating strategy was provided in the supplemental data.

☒ Tick this box to confirm that a figure exemplifying the gating strategy is provided in the Supplementary Information.
